# Beta cell dedifferentiation in type 1 diabetes: sacrificing function for survival?

**DOI:** 10.3389/fendo.2024.1427723

**Published:** 2024-06-06

**Authors:** Kierstin L. Webster, Raghavendra G. Mirmira

**Affiliations:** Kovler Diabetes Center and the Department of Medicine, The University of Chicago, Chicago, IL, United States

**Keywords:** type 1 diabetes, beta cell, dedifferentiation, autoimmunity, islet

## Abstract

The pathogeneses of type 1 and type 2 diabetes involve the progressive loss of functional beta cell mass, primarily attributed to cellular demise and/or dedifferentiation. While the scientific community has devoted significant attention to unraveling beta cell dedifferentiation in type 2 diabetes, its significance in type 1 diabetes remains relatively unexplored. This perspective article critically analyzes the existing evidence for beta cell dedifferentiation in type 1 diabetes, emphasizing its potential to reduce beta cell autoimmunity. Drawing from recent advancements in both human studies and animal models, we present beta cell identity as a promising target for managing type 1 diabetes. We posit that a better understanding of the mechanisms of beta cell dedifferentiation in type 1 diabetes is key to pioneering interventions that balance beta cell function and immunogenicity.

## Introduction

Type 1 and type 2 diabetes (T1D and T2D) are characterized by a loss of functional pancreatic beta cell mass. In T1D, the loss of beta cell mass has largely been attributed to autoimmune-mediated killing of beta cells, whereas in T2D, the loss of beta cells has been attributed to dedifferentiation—a process by which cells lose some or all of their specialized features. The emerging picture suggests, however, that beta cell death and dedifferentiation each contribute to the pathogenesis of both type 1 and type 2 diabetes.

T1D is conventionally defined as a disorder of immune tolerance, yet it is increasingly appreciated that beta cell dysfunction precedes T1D onset and that beta cells themselves may play a central role in the pathogenesis of the disease. Although the term ‘dedifferentiation’ has been used less often in the context of T1D, studies in human tissues and mouse models indicate that beta cell identity is indeed altered in T1D and that this may have critical implications for how beta cells interact with the immune system. In this perspective, we will consider the concept of beta cell identity, summarize the evidence for beta cell dedifferentiation in T1D, and speculate how dedifferentiation might influence beta cell susceptibility to autoimmunity.

## What is beta cell identity?

Pancreatic beta cells are endocrine cells in the islets of Langerhans that synthesize and secrete insulin to control blood glucose. Mature beta cell function requires the ability to sense blood glucose levels quickly and accurately, the biosynthetic capacity to mass produce and process insulin, the dexterity to fine-tune its release, and coordination with other islet cells to avoid over- or under-responding to glucose flux. A significant effort to date has shed light on how ‘beta cell identity’—the unique repertoire of cellular proteins that equip beta cells for these tasks—is developed and maintained *in vivo* and how alterations in beta cell identity may be a consequence or even cause of diabetes (1–4).

Beyond insulin production, beta cell identity is often assessed through the production of beta cell-enriched transcription factors such as Pdx1, MafA, Foxo1 and Nkx6.1. Together, these transcription factors guide the differentiation and maturation of beta cells and maintain mature beta cell function in adulthood by regulating genes related to glucose sensing and transport (e.g., *Slc2a2, Gck*), mitochondrial function (e.g., *Mfn1/2, Drp1*), calcium signaling (e.g., *Ryr2, Serca2b*), and insulin production (e.g., *Mafa, Pdx1, Ins1/2*) (5–8). Absence of *Pdx1* during murine and human development, for example, leads to complete pancreatic agenesis (9, 10), while beta cell specific *Pdx1* depletion in adult mice results in loss of insulin expression, downregulation of *MafA*, and rapid onset hyperglycemia (11). Heterozygous mutations in *Pdx1* in humans lead to a form of monogenic diabetes termed maturity-onset diabetes of the young 4 (MODY4) (12).

Beta cell identity also relies upon the *repression* of other cellular programs, as shown through inducible knockouts of enriched transcription factors in mice. Ablation of *Pdx1* derepresses alpha cell transcription factor MafB in adult beta cells, manifesting a phenotype closely resembling alpha cells (11), and *Nkx6-1* deletion leads embryonic or adult beta cells to co-express insulin and somatostatin (13). At least 60 ‘disallowed’ genes have been identified in beta cells (14–16), many relating to the sensing of glucose and coupling of its metabolism to insulin exocytosis. For example, beta cells repress the expression of high-affinity hexokinases in favor of low-affinity glucokinase to avoid insulin release at low blood glucose levels (17, 18). Similarly, low monocarboxylate transporter 1 (MCT-1) expression prevents beta cells from taking up circulating lactate or pyruvate (i.e. during exercise) (19, 20), and low lactate dehydrogenase A (LDHA) prevents interconversion of these metabolites and their entry into the TCA cycle (21, 22). Also among the beta cell repressed genes are cell proliferation factors, such as *Pdgfra, c-Maf*, and *Igfbp4* (16), whose repression likely contributes to the largely quiescent state of mature beta cells. Selective gene repression is controlled by inhibitory actions of the beta cell transcription factors (11, 23), epigenetic modifications (24, 25), and by microRNAs and long noncoding RNAs (26–28).

Importantly, beta cell identity is neither uniform nor static. Single-cell RNA sequencing (scRNA-Seq) and electrophysiological studies reveal subpopulations of beta cells that exhibit different abundances, transcriptional signatures, maturity levels, and glucose response dynamics even within the same islet (29). Disentangling the roles of these subpopulations, how they interact, and how they shift during diabetes pathogenesis may enable us to manipulate the state of beta cell identity as a therapeutic avenue.

## What is beta cell dedifferentiation?

Cellular dedifferentiation is the process by which a mature cell loses some or all of its specialized features. Through dedifferentiation, a terminally differentiated cell may re-express markers of lineage precursors, re-enter the cell cycle, and proliferate. Controlled dedifferentiation plays a physiological role in the remarkable tissue regeneration abilities of other species and in the more limited regenerative capacity of mammalian cardiac and nervous tissues after injury (30). In cancer, conversely, dedifferentiation of tumor cells is associated with increased metastatic potential, drug resistance, and evasion of immune surveillance (31).

Dedifferentiation of the pancreatic beta cell has been observed in models of metabolic stress. Rat studies demonstrated that beta cells decrease their expression of beta cell transcription factors *Pdx1*, *Pax6*, and *Nkx6-1* and increase expression of ‘disallowed’ genes like *Ldha* and *Hk1* in response to partial pancreatectomy and resultant hyperglycemia (32). Later, lineage tracing experiments in mice showed that *Foxo1* depletion causes hyperglycemia, driven by beta cell dedifferentiation and transdifferentiation to alpha cells (33). Intriguingly, these same processes have been observed in *db/db* and GIRKO mouse models of T2D (33) as well as in diet-induced obesity models (34, 35), prompting interest in whether beta cell dedifferentiation promotes diabetes in humans.

## What has been seen in T2D?

Studies of pancreatic tissue from organ donors with T2D suggest that beta cell identity is altered compared to nondiabetic donors. The number of islet cells with endocrine features (i.e., expression of synaptophysin or chromogranin A) is maintained in T2D donors, yet the number of insulin-positive cells is profoundly decreased, and glucagon-positive cells increased (36, 37). Some studies report an increase in insulin and glucagon double-positive cells in T2D donors (37, 38), while others find no difference compared to nondiabetic donors (36). Further, expression of aldehyde dehydrogenase 1a3 (ALDH1A3), which marks failing and dedifferentiated beta cells across several mouse models of T2D (39, 40), is significantly increased in pancreata from human donors with T2D (37). These data suggest that endocrine cell *death* cannot adequately explain the loss of functional beta cell mass in T2D.

Beyond just hormone markers, scRNAseq and complementary approaches in mice and humans have begun to more comprehensively define the transcriptional state of beta cell identity at baseline and in T2D. For example, a Cd63^high^ cluster of beta cells with high levels of mitochondrial metabolism and glucose-stimulated insulin secretion — characteristic of mature beta cells — was found to be markedly reduced in mouse and human T2D (41). Conversely, Cd81^high^ beta cells (expressing low levels of *Mafa, Ucn3*, and *Glut2*) are a more immature cluster, whose abundance is increased in mouse models of T2D and human islets subjected to ER or glucolipotoxic stress *in vitro* (42). A study cataloging human beta cell gene expression patterns across ages from newborn to adult found that in adult donors with T2D, a ‘newborn’ pattern of expression reemerged (43). These studies demonstrate a shift away from mature beta cell phenotypes in T2D. This shift has been postulated as a ‘selfish’ survival strategy—an attempt by stressed beta cells to ‘rest’ and avoid exhaustion and apoptosis (33).

## What has been seen in T1D?

T2D and T1D are both associated with loss of functional beta cell mass, though traditionally, T2D is associated with beta cell dysfunction and T1D with beta cell death. The features of T2D thought to drive beta cell dedifferentiation, such as hyperglycemia and proinflammatory signaling (32, 44), are also features of T1D. Whether or not beta cells dedifferentiate in T1D has not been extensively explored.

Studies of pancreas from T1D donors indicate altered endocrine cell identity. In T1D, there is a profound loss of insulin-positive cells in the islets (45). Even in longstanding T1D, however, some insulin-positive residual beta cells remain (46, 47), and proinsulin is detectable in both pancreata and sera of individuals with T1D for years after disease onset (48, 49). What allows some beta cells to persist is unclear. While it is thought that the loss of beta cell mass in T1D is primarily due to cell death, there is limited direct evidence of this killing in human tissues (45). Although beta cells typically comprise about 50–65% of human islet volume in healthy individuals (50), there is no apparent decrease in islet size in T1D donors (51). Notably, there is a relative increase in non-beta endocrine cell types (52). Insulin-deficient T1D islets consist largely of cells co-producing glucagon and Pdx1 (51), a sign of possible transit between beta and alpha cell identities. Like in T2D, endocrine cells expressing none of the 4 major pancreatic hormones (ChrgA+/hormone-, or CPHN) are also more abundant in T1D islets than in nondiabetic or autoantibody-positive (Aab+) islets (52). The source of these CPHN cells is undetermined. Collectively, these data raise the possibility that while some beta cells are lost to immune-mediated killing in T1D, some dedifferentiate to phenotypes expressing little to no insulin, such as CPHN or other endocrine cell types.

## What is driving beta cell dedifferentiation in T1D?

Human T1D pancreas tissues are a scarce resource, and one that only captures a limited cross-section of disease pathology. Therefore, other *in vivo* and *in vitro* models have been primarily used to study the mechanisms and functional consequences of beta cell dedifferentiation in T1D. Nonobese diabetic (NOD) mice develop immune infiltration of the islet (insulitis), as well as beta cell endoplasmic reticulum stress, by about 4–8 weeks of age; however, most mice will not develop overt autoimmune diabetes until 12–20 weeks of age (53). By 8 weeks of age, there is a significant decrease in islet expression of *Pdx1* and of *Ins1/2* (53) without a decrease in beta cell mass (54). This finding could represent a program of beta cell dedifferentiation either intrinsic to beta cells on the NOD background or spurred by increasing islet inflammation. *In vitro* studies support the association of inflammation and beta cell dedifferentiation; human, rat, and mouse islets treated with proinflammatory cytokines show decreases in beta cell genes like *Pdx1*, *MafA*, and *Nkx6–1* (44, 55). Notably, NOD-SCID mice are B and T cell-deficient and do not develop spontaneous autoimmune diabetes, yet they also exhibit a decrease in islet *Pdx1* and *Ins1/2* expression with age (53). IL-1b appears to be particularly potent in downregulating beta cell markers (44), and this cytokine is primarily secreted by innate immune cells, which remain intact in NOD-SCID mice.

Examining NOD islets at the single-cell level has elaborated on the dedifferentiation phenomenon. Starting around 4 weeks, a population of beta cells with decreased insulin content and maturity markers yet increased PD-L1 and markers of stemness (‘Btm’ beta cells) appears, their proportion rising as they preferentially survive immune infiltration (56). While hyperglycemia is strongly associated with dedifferentiation in other disease models (32, 57), Btm beta cells appear before hyperglycemia in the NOD model. PD-L1+ beta cell abundance correlates with increasing abundance of CD45+ cells in the islet (56). Blocking T cell killing of beta cells via administration of an anti-CD3 monoclonal antibody decreased but did not eliminate the formation of Btm beta cells, suggesting that islet infiltrating T cells contribute to but cannot entirely account for the formation of these immature beta cells.

The above studies suggest that dedifferentiation is part of the ‘natural history’ of NOD diabetes. Other studies have identified beta cell dedifferentiation in response to genetic and pharmacological inhibition of essential beta cell functions, providing clues for how inflammation or intrinsic beta cell defects may activate this process in T1D. Exposing the human beta cell line EndoC-betaH1 to double-stranded RNA to simulate viral infection, a possible trigger for T1D initiation (58), causes reductions in genes like *MAFA* and *INS* and increases in progenitor markers like *SOX9, HES1*, and *MYC* driven by NF-kB within the beta cell, as well as by interferon alpha released by neighboring cells (59, 60). *Endogenous* double-stranded RNAs- allowed to persist in the setting of beta cell-specific depletion of RNA editing enzyme ADAR- decrease proprotein convertase expression and formation of mature insulin, and also elicit massive interferon alpha responses and insulitis (61). Importantly, high glucose also exacerbates the interferon response, creating a positive feedback loop that may be acting in early T1D- interferons decrease beta cell functionality, and decreased functionality leads to poorer glucose handling (61). Oxidative stress also decreases maturity genes and increases progenitor markers in primary human beta cells (62). Directly reactivating developmental pathways, such as Notch or Hedgehog signaling, in mature mouse beta cells stimulates proliferation (63, 64). Beta cell-specific depletion of mTORC component Raptor in mice disrupts mitochondrial metabolism but also decreases beta cell-enriched genes and increases progenitor markers in a hyperglycemia-independent manner (65). Disruption of the unfolded protein response (UPR), which is activated by ER stress in prediabetic NOD mice (53), causes beta cell dedifferentiation in models of both T1D and T2D (66, 67). Studies in the murine beta cell line MIN6 suggest that loss of beta cell maturity induced by even near-fatal levels of ER stress is reversible, yet plasticity decreases with repeated episodes of ER stress (68). Thus, developing strategies to intervene before the potential for beta cell redifferentiation is lost may be important to recovering beta cell function long-term.

Studies across different models of T2D (*ob/ob*), T1D (NOD), and inflammatory beta cell death (metronidazole) each demonstrate beta cell dedifferentiation phenotypes, none of which perfectly recapitulate a simple reversion to beta cell progenitor states (69–71). Importantly, heterogeneity between these transcriptomic and functional phenotypes stresses that beta cell dedifferentiation is not a single linear process, but rather varies to the particular diabetogenic stressors at hand. Further study will be needed to understand which of the above pathways (i.e. interferon signaling, ER stress, reactivation of developmental pathways) are most relevant to human T1D and at what stages of disease.

## How does dedifferentiation impact beta cell-directed autoimmunity?

Although evidence of beta cell dedifferentiation in T1D continues to emerge, it is unclear what role this phenomenon plays in disease pathophysiology. A loss of mature beta cell identity often means a loss in effective glucose-stimulated insulin secretion, yet these immature cells appear to preferentially survive autoimmunity. As noted previously, dedifferentiation of tumor cells is associated with increased metastatic potential, drug resistance, and evasion of immune surveillance (31). While these are dangerous features in cancer, could dedifferentiation also be a strategy by which beta cells evade autoimmune killing?

**Figure 1:**
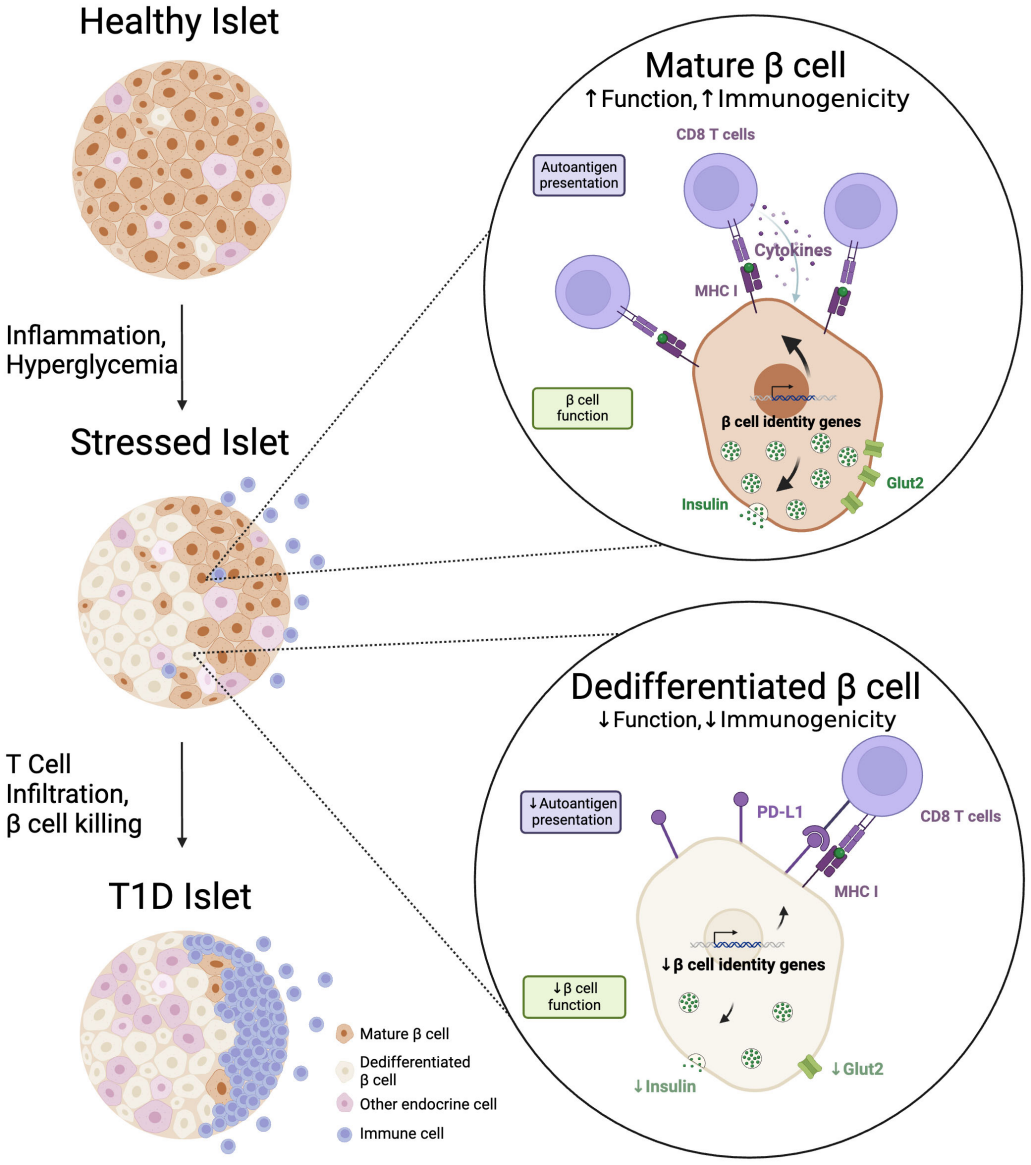
Dedifferentiated beta cells preferentially survive autoimmune attack. During the progression to T1D, an increasing proportion of beta cells exhibit an immature or 'dedifferentiated' phenotype. We propose that dedifferentiation decreases beta cell functional characteristics (i.e. expression of insulin, Glut2), but that this decrease, along with augmentation of immune checkpoint proteins like PD-L1, also serves to decrease beta cell immunogenicity in the autoimmune setting of T1D. Created with BioRender.com.

The resilient (‘Btm’) beta cells described by Rui et al. in NOD mice express lower levels of beta cell autoantigens IGRP, ZnT8, Gad1, and IA-2 compared to their more susceptible counterparts (‘Top’), while also expressing higher levels of immune tolerogenic proteins PD-L1 and Qa-2 (56). These immunoprotective features of the Btm beta cells offer a strong defense against immune killing in spontaneous NOD diabetes, cyclophosphamide-induced diabetes, and *in vitro* in a culture of sorted Top and Btm cells with islet immune infiltrates. Several models in which beta cell identity is altered on the NOD background also protect against T1D-like disease. Inducing early beta cell-specific knockout of UPR protein IRE1-α (IRE1-α^beta-/-^) causes a phenotype similar to the Btm beta cells (i.e. decreased autoantigens, increased immunoinhibitory markers), as well as decreased MHC class I and peptide loading components (67). These mice become transiently hyperglycemic but ultimately recover and are protected from diabetes. During the hyperglycemic phase, IRE1-α^beta-/-^ islets show decreased insulin expression; during the recovery period, these islets have less immune infiltration compared to controls and recovered insulin content. These findings are consistent with a hypothesis that stressed beta cells use dedifferentiation to ‘rest’ and dampen pro-apoptotic interactions with the immune system (**Figure 1**) and suggest that these cells may be capable of redifferentiation following acute stress. Other mouse models are also consistent with this hypothesis, including NOD Liver Insulin Receptor knockout (LIRKO) mice and early beta cell knockout of the UPR protein ATF6 in NOD mice —each induces transient beta cell dysfunction followed by protection from T1D. The NOD-LIRKO model, characterized by proliferating beta cells bearing fewer autoantigens, exhibits only transient hyperglycemia and is protected by an increase in regulatory T cells. Transplanted into NOD mice, NOD-LIRKO islet grafts showed improved survival compared to control islets, indicating islet-intrinsic protection that is likely afforded by the presence of immature, proliferative beta cells (72). In the case of ATF6 KO induced shortly after birth (postnatal days 1–3), beta cell induction of the senescence associated secretory phenotype (SASP)- increased secretion of select cytokines, chemokines, and growth factors- protected NOD mice from diabetes by recruiting inflammation-resolving M2 macrophages to the islet (73). Like dedifferentiation, *senescence* represents an altered state of beta cell identity which has been observed in human T1D islets and is associated with decreased expression of maturity markers like Ucn3 (74). In contrast to dedifferentiated cells, which often show increased markers of proliferation and stemness (56), senescent cells exit the cell cycle. Interestingly, while induction of senescence prior to insulitis protects NOD mice from developing diabetes (73), selective *clearance* of senescent beta cells in adult NOD mice after insulitis also reduces diabetes development (74). This seeming contradiction underscores that alteration of beta cell identity may be protective or detrimental depending on timing and disease context, with immature features showing particular potential to protect beta cells from the damages of insulitis.

Whereas the relationship between beta cell identity and autoimmunity is more difficult to study directly in human disease, T1D tissues offer some parallels to observations in mice. Like Btm cells in NOD mice, remaining insulin-positive cells in T1D islets express PD-L1, which is absent in nondiabetic, AAb+, and even insulin-negative T1D islets (75). Single-cell initiatives (e.g., the Human Pancreas Analysis Program and spatial transcriptomics and proteomics) will help determine if these PD-L1+ residual beta cells, or the CPHN or Pdx1+-Glucagon+ cells observed in human T1D islets, behave like the resilient beta cells observed in NOD mice, and perhaps how they relate to proximity or composition of insulitis.

## Discussion

An accumulating body of evidence links both T1D and T2D pathogenesis to the emergence of dedifferentiated beta cells with loss of hormone expression, expression of markers from multiple endocrine cell types, and re-expression of progenitor genes. Inflammatory signaling and hyperglycemia, hallmarks of both T1D and T2D, are known to downregulate beta cell maturity markers. Beta cell dedifferentiation has conventionally been viewed as a purely detrimental process through which beta cells lose the ability to produce and secrete insulin in response to glucose, culminating in functional failure. Nonetheless, the universal detrimental implications of beta cell dedifferentiation remain speculative, especially within the context of autoimmune T1D.

Sophisticated single-cell analyses conducted in both human and murine models have shown the transcriptional and functional heterogeneity of beta cells, revealing subpopulations capable of withstanding immune onslaught. Notably, in the NOD mouse model of T1D, beta cells expressing fewer autoantigens and augmented immune checkpoint proteins, either spontaneously or through genetic interventions, tend to persist. We propose that beta cells may dedifferentiate into immature phenotypes to evade immune surveillance, albeit at the expense of their functionality (**Figure 1**). Thus, dedifferentiation may also serve to alleviate the biosynthetic burdens on beta cells and promote ‘beta cell rest’ – a clinical concept that explains why suppressing endogenous insulin secretion with exogenous insulin in newly-diagnosed T1D patients ultimately reduces their long-term exogenous insulin requirements (76).

In individuals at risk of developing T1D, establishing a homeostatic equilibrium between diminished functionality and attenuated immune recognition of beta cells may be an avenue for therapeutic intervention. Future investigations are warranted to determine the feasibility of pharmacologically inducing beta cell dedifferentiation or redifferentiation. Functional targets (i.e. ER stress, RNA editing), tissue specificity, and timing of therapy relative to disease pathogenesis will be key areas for exploration.

## Data availability statement

The original contributions presented in the study are included in the article/supplementary material. Further inquiries can be directed to the corresponding author.

## Author contributions

KW: Conceptualization, Writing – original draft, Writing – review & editing. RM: Conceptualization, Writing – review & editing, Funding acquisition.
